# UST-YOLO11Pose-TRM: An Attention-Enhanced Keypoint Detection and Transformer Regression Framework for Yak Body Measurement

**DOI:** 10.3390/ani16101493

**Published:** 2026-05-13

**Authors:** Hua Li, Jinghan Cai, Tonghai Liu, Yapeng Xiao, Changran Liu, Can Zhou

**Affiliations:** 1 College of Computer and Information Engineering, Tianjin Agricultural University, Tianjin 300392, China; l1882208@126.com (H.L.); q1463747994@163.com (J.C.); xypstudy1@163.com (Y.X.); liuchangran715@163.com (C.L.); 15136793760@163.com (C.Z.); 2 College of Engineering and Technology, Tianjin Agricultural University, Tianjin 300392, China

**Keywords:** yak body measurement, keypoint detection, UST-YOLO11Pose-TRM, transformer regression, smart farming

## Abstract

Yak (Bos grunniens) is a vital livestock resource on the Qinghai–Tibet Plateau, and its body measurement parameters play a crucial role in growth and development assessment, health monitoring, and breeding improvement. To overcome the limitations of traditional manual measurements—such as low efficiency, unstable accuracy, and the tendency to induce animal stress—this study proposes an intelligent yak body measurement prediction method that integrates keypoint detection with regression modeling, termed UST-YOLO11Pose-TRM. Within the YOLO11-Pose framework, three attention mechanisms—UIB, SENetV2, and TripleAttention—are incorporated to construct a lightweight yet high-precision keypoint detection model, UST-YOLO11Pose, thereby enhancing channel feature representation, global contextual modeling, and spatial dependency perception. Meanwhile, a Transformer-based regression model is designed, leveraging multi-head self-attention to characterize global geometric relationships among keypoints and to achieve accurate prediction of key body measurement parameters, including body length, body height, oblique body length, chest girth, and cannon circumference. Experimental results demonstrate that UST-YOLO11Pose achieves an mAP of 0.958, a Precision of 0.967, and a Recall of 0.955 in keypoint detection tasks, significantly outperforming both same-series and cross-series comparative models with a parameter size of only 10.06 MB. In the body measurement regression task, the Transformer-based regression model attains an RMSE of 0.185, an MAE of 0.122, an MAPE of 2.3%, and a coefficient of determination (R^2^) of 0.962 on the test set, indicating excellent predictive accuracy and robust fitting stability. In summary, UST-YOLO11Pose-TRM enables accurate, efficient, non-contact yak body measurement, showing strong potential for smart pasture development and precision livestock management.

## Simple Summary

This study addresses the challenge of measuring yak body size efficiently and accurately, as traditional manual methods are time-consuming, error-prone, and stressful for animals. The objective is to develop a non-contact, intelligent measurement approach using computer vision and deep learning. To achieve this, this study proposes a framework that first detects key body points on yak images and then predicts body measurements using an advanced regression model. The results show that the method achieves very high accuracy in both keypoint detection and measurement prediction, outperforming existing models while remaining lightweight and fast. The study concludes that this approach can reliably estimate key body parameters without physical contact. This has important practical value, as it can improve animal welfare, reduce labor, and support precision livestock management, contributing to more efficient and sustainable farming systems in harsh plateau environments.

## 1. Introduction

The yak (Bos grunniens) is a rare livestock species endemic to the Qinghai–Tibetan Plateau and surrounding high-altitude regions. It has fully adapted to extreme environmental conditions such as low temperatures, hypoxia, and strong ultraviolet radiation, and serves as a vital means of livelihood for local ethnic minorities, particularly Tibetan communities [1]. By providing meat, milk, and fiber, and by contributing to the maintenance of ecological balance, yaks play an irreplaceable role in high-altitude pastoral systems [2,3]. Tongren City in the Huangnan Tibetan Autonomous Prefecture of Qinghai Province is one of the major yak-breeding areas, where the Xiabulang Village Cooperative has a long history of yak husbandry. However, constrained by the complex plateau terrain and traditional breeding practices, yak growth monitoring still relies predominantly on manual measurement, resulting in low efficiency and accuracy that is highly dependent on operator experience [4,5].

In yak breeding and management, body measurement parameters—such as body length, body height, oblique body length, chest girth, and cannon bone girth—are core indicators for evaluating growth and development, health status, reproductive performance, and meat production potential [6]. Accurate acquisition of these parameters is of great importance for optimizing feeding and management strategies, selecting superior breeds, and improving overall production efficiency [7,8]. For example, chest girth is strongly correlated with body weight and can be used to rapidly assess growth status, while body height and body length directly reflect body conformation development and provide critical references for genetic improvement and breeding [8]. Traditional yak body measurement relies on manual operations, requiring direct contact with animals using tools such as measuring tapes and measuring sticks [6]. This approach is not only time-consuming and labor-intensive, but also prone to inducing stress responses in yaks, which can increase measurement errors [9]. It is difficult to meet the demands of high-frequency monitoring in large-scale breeding and management systems. Moreover, yaks are predominantly distributed in high-altitude and cold regions such as the Qinghai–Tibetan Plateau, where harsh environmental conditions further exacerbate the difficulty of manual measurements [5,10]. Therefore, the development of efficient, non-contact body measurement technologies has become a key direction for advancing intelligent yak breeding and management.

In recent years, advances in computer vision (CV) and deep learning have provided new technological pathways for the automated measurement of livestock body dimensions. Image-based, non-contact approaches estimate body size by extracting surface features such as keypoints, contours, or segmentation regions, and have been extensively validated in livestock including cattle and pigs [6]. For yaks, a plateau-specific species, several studies have employed YOLO-series models to perform posture recognition, body surface parameter extraction, and body weight estimation, demonstrating the feasibility and application potential of such models for non-contact yak body quantification tasks [11,12,13]. Further studies indicate that YOLO-based models exhibit relatively strong robustness under complex natural environments typical of yak habitats; however, factors such as dense hair occlusion, large posture variations, and unstable lighting conditions still significantly affect the accuracy of body surface feature extraction [2,11]. However, most existing studies mainly focus on improving detection performance at the model level while paying relatively limited attention to the robustness of keypoint localization under extreme plateau conditions. In particular, the interaction between occlusion, posture variation, and lighting disturbances is not explicitly modeled, which may lead to unstable feature extraction results.

To further improve the detection performance of YOLO-series models in complex livestock farming scenarios, extensive research in recent years has focused on the introduction of attention mechanisms and network lightweight optimization. In body surface analysis tasks involving cattle, yaks, and other large livestock, integrating channel attention, spatial attention, or multi-scale feature enhancement modules into the YOLO framework has been shown to effectively enhance the model’s ability to perceive local key structures (such as the head, limbs, and trunk contours) while improving detection stability in complex backgrounds with relatively low computational overhead [14,15,16,17]. In addition, YOLO variants that combine lightweight convolutional architectures with improved feature pyramid networks have demonstrated a favorable balance between real-time performance and detection accuracy in dense individual detection and edge-device deployment scenarios [18,19], providing strong technical support for practical applications in intelligent livestock farming under plateau pastoral conditions. Nevertheless, these improvements are primarily designed for general object detection tasks, and their effectiveness in fine-grained keypoint localization for livestock body measurement remains insufficiently explored. In particular, the lack of task-specific optimization for yak morphological characteristics may limit their practical performance.

At the level of body measurement parameter prediction, most existing studies adopt a two-stage paradigm of “keypoint detection + regression modeling.” Specifically, models such as YOLO-Pose are first used to obtain the coordinates of animal body surface keypoints, after which regression models are employed to learn the geometric relationships among these keypoints for body size or body weight prediction. Related studies have shown that models such as multilayer perceptrons (MLPs) and long short-term memory networks (LSTMs) can, to some extent, capture the nonlinear mapping between body measurement parameters and keypoint features [20,21]; however, their ability to model global geometric structures remains limited. In recent years, Transformer models based on self-attention mechanisms have gradually been introduced into livestock body size and weight prediction tasks due to their advantages in modeling global feature dependencies, demonstrating stronger structural representation capabilities and generalization potential [22,23]. However, the application of Transformer-based models in livestock body measurement is still at an early stage, and their advantages in modeling complex geometric relationships among keypoints have not been fully exploited. In addition, existing studies rarely integrate advanced detection models with Transformer-based regression in a unified framework.

However, in yak body size prediction tasks, issues such as the amplification of keypoint localization errors and the inadequate modeling of complex body conformation structures remain unresolved. How to construct an end-to-end prediction framework that simultaneously balances detection accuracy and regression stability therefore still requires further investigation. First, yaks exhibit distinctive morphological characteristics, such as dense hair and robust limbs, together with large movement amplitudes, which limit the accuracy of existing keypoint detection models (e.g., YOLOv7-pose) in complex environments [2]. Second, most existing studies rely on a single detection model or regression algorithm, lacking systematic comparisons of different model architectures and tailored data augmentation strategies for yak body size characteristics [4,5]. Third, in plateau grazing environments, yaks frequently adopt non-standard postures such as head-down grazing or lateral resting, and the strong ultraviolet radiation at high altitudes further induces image distortions, jointly leading to degraded keypoint localization accuracy that has not been specifically addressed in previous work [24]. Finally, certain yak body size parameters (e.g., oblique body length and cannon circumference) have unique geometric definitions that typically require joint modeling from multi-view images (e.g., top-view and side-view), whereas few existing methods rely solely on single-view data [8]. In summary, existing studies still face limitations in both robust keypoint detection under complex plateau conditions and the effective modeling of global geometric relationships among body features. These challenges highlight the need for an integrated framework that can simultaneously enhance feature representation and capture long-range dependencies, which motivates the present study.

To address the above challenges, the objective of this study is to develop and evaluate an accurate and robust non-contact yak body measurement framework based on deep learning. Specifically, this work aims to improve keypoint detection performance under complex plateau conditions and enhance the modeling of geometric relationships among body measurement features.

To achieve this, an improved keypoint detection model integrating multiple attention mechanisms is designed to enhance feature representation and localization accuracy. Furthermore, a Transformer-based regression model is introduced to effectively capture global dependencies and complex geometric relationships among keypoints, enabling more accurate estimation of body measurements. By combining these components, the proposed framework provides an integrated solution for precise and efficient non-contact livestock measurement.

The main contributions of this study are summarized as follows:

(1) A lightweight and robust yak keypoint detection model, UST-YOLO11Pose, is developed by integrating UIB, SENetV2, and TripleAttention into the YOLO11-Pose framework. This design enhances feature representation, channel interaction, and spatial attention, significantly improving keypoint localization accuracy under complex plateau conditions such as occlusion, posture variation, and background interference.
(2) A Transformer-based regression model is introduced for yak body measurement estimation. By leveraging multi-head self-attention, the model explicitly captures global geometric dependencies among keypoint-derived features, overcoming the limitations of conventional regression methods (e.g., MLP, CNN, and tree-based models) in modeling long-range spatial relationships.
(3) An end-to-end non-contact measurement framework (UST-YOLO11Pose-TRM) is constructed, which effectively integrates keypoint detection and regression modeling into a unified pipeline. This framework achieves a balance between prediction accuracy and computational efficiency, making it suitable for practical deployment in plateau pasture environments.
(4) Comprehensive experiments, including model comparisons, ablation studies, and generalization evaluations, are conducted to systematically validate the effectiveness, robustness, and practical applicability of the proposed method.

## 2. Materials and Methods

### 2.1. Data Acquisition

Data collection for this study was conducted from April to May and from July to August 2025 at the Xiabulang Village Cooperative in Tongren City, Huangnan Tibetan Autonomous Prefecture, Qinghai Province, China. This region represents a typical plateau pastoral area characterized by a complex natural environment and extensive experience in yak husbandry, thereby providing a representative and reliable data source for research on yak body measurement prediction methods.

To ensure high image quality and measurement accuracy, a Sony A7M2 digital camera (24.3-megapixel effective resolutionmanufactured by Sony Corporation in Chonburi, Thailand.) was used to capture images of yaks. During side-view acquisition, the camera was mounted on a tripod at a height of 0.7 m above the ground to ensure imaging under the yak’s natural standing posture and to minimize geometric distortion caused by viewpoint deviations. For top-view acquisition, a 2 m-high imaging platform was constructed, allowing the camera to capture images vertically downward. All images were collected under natural lighting conditions to avoid interference from shadows and color inconsistencies introduced by artificial illumination. All images were collected under natural plateau grazing conditions, covering a range of real-world scenarios. These include variations in illumination (e.g., strong sunlight, partial shadow, and backlighting), moderate background complexity, and natural posture changes during grazing and standing. In addition, partial occlusion caused by body parts (e.g., head, limbs, and torso overlap) was present in some samples due to natural movement and camera viewpoint. However, it should be noted that large-scale herd-level occlusion (e.g., dense multi-individual overlap) and extreme weather-induced lighting conditions were relatively limited in the current dataset. Therefore, while the dataset captures common individual-level variations in plateau pasture environments, it does not yet fully cover all high-frequency complex scenarios. Although keypoint detection and body measurement modeling in this study were primarily performed using side-view images, a small number of top-view images were additionally collected during the early data acquisition stage to enhance the completeness of the data collection scheme and to provide a foundation for future multi-view research. It should be noted that all experimental results reported in this paper are based exclusively on side-view data, and the top-view images were not involved in the core modeling process of this study.

To align keypoint coordinates with real-world physical scales, a 1.1 m measuring ruler was placed on the ground within the same imaging plane as the yak before each shooting session, serving as a scale reference. In addition, a unique individual ID was assigned to each yak to ensure data traceability and annotation consistency. In total, image data from 111 yaks were collected, with each individual represented by three side-view images. While maintaining consistent camera height and shooting distance, natural minor variations in head posture and limb stance were allowed across the three side-view images to enhance pose diversity. This resulted in a total of 333 raw images.

After image acquisition, body measurements were conducted for each yak by two experienced technicians. The measured parameters included body length, body height, oblique body length, chest girth, and cannon circumference. To reduce human-induced errors, each parameter was measured three times, and the average value was taken as the ground-truth body measurement. Meanwhile, body weight information for each individual was recorded using an electronic scale, which was used to assist in validating the rational relationship between body measurements and growth status, and to provide a reference for subsequent studies on body-weight prediction based on body measurements. This standardized data acquisition procedure provides a reliable data foundation for subsequent precise keypoint annotation and model training.

The collected dataset includes yaks of varying body sizes and growth conditions to reflect a certain degree of population variability. However, detailed demographic information such as age, gender, and breed subtype was not systematically recorded during data acquisition due to practical constraints in field conditions. As a result, the dataset primarily reflects phenotypic diversity observable from body morphology rather than explicitly controlled biological attributes.

### 2.2. Data Preprocessing

To ensure the reliability and consistency of keypoint detection and regression modeling in the yak body measurement prediction task, the raw image data and corresponding body measurement values were systematically preprocessed. The main preprocessing steps included keypoint annotation, scale calibration, data augmentation, dataset partitioning, and normalization.

**Figure 1:**
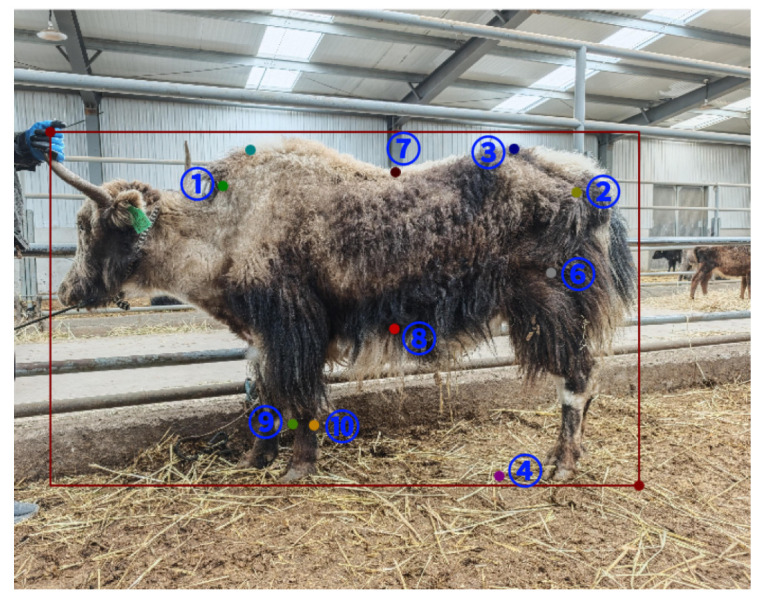
Annotation example of body measurement keypoints on yak images.

First, all images were manually annotated using the open-source annotation tool Labelme (version 6.0.2). During the annotation process, the contour of the yak body was delineated first, followed by the labeling of keypoints at anatomically significant locations on the yak surface (as shown in Figure 1). A total of 10 keypoints were annotated, including the shoulder point, hip point, knee joint, hoof point, and intersections along the dorsal line, which were used for subsequent calculation of body measurement features such as body length, oblique body length, body height, chest girth, and cannon circumference. All samples were annotated by the author under the guidance of experienced annotators, and consistency checks were performed to reduce human annotation bias, thereby ensuring the stability and reliability of the annotation results.

Before keypoint detection and distance computation, geometric calibration was performed on the keypoint coordinates using the 1.1 m reference ruler placed during image acquisition in order to establish a mapping between pixel coordinates and real-world physical scales. Subsequently, Euclidean distances were calculated based on the annotated two-dimensional keypoint coordinates to construct distance vectors that describe the geometric structural characteristics of the yak body surface. These distance vectors were then matched one-to-one with the ground-truth body measurement parameters obtained from manual measurements—namely body length, body height, oblique body length, chest girth, and cannon circumference—for subsequent regression modeling.

**Figure 2:**
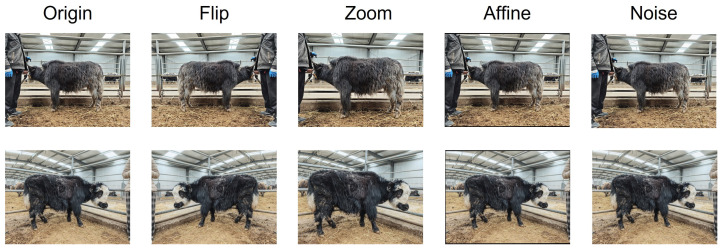
Examples of data augmentation strategies applied to the training images.

To alleviate the limitation of a small sample size and enhance the robustness of the model to varying shooting conditions and environmental changes, data augmentation was applied exclusively to the training set samples (as illustrated in Figure 2). The specific augmentation strategies included horizontal flipping, random scaling with a ratio range of 0.8–1.2, affine transformations (rotation angles of ±15° and translation up to ±10%), and the addition of Gaussian noise. Through these augmentation operations, the dataset size was expanded from the original 333 images to 1080 images, significantly increasing sample diversity.

During dataset partitioning, the data were split based on individual yak IDs rather than randomly at the image level. The dataset was divided into training, validation, and test sets at a ratio of 7:2:1, ensuring that images of the same yak did not appear in multiple subsets. Before splitting, the dataset was randomly shuffled to ensure that samples were uniformly distributed across subsets and to reduce potential bias. This strategy effectively prevents information leakage and enhances the fairness and credibility of the model evaluation results.

In addition, to meet the input requirements of the improved YOLO11-Pose keypoint detection model integrating the UIB, SENetV2, and TripleAttention modules, all images were uniformly resized to 640 × 640 pixels and subjected to pixel-value normalization. The corresponding keypoint coordinates and distance features were normalized accordingly to ensure consistency in numerical distributions across different feature scales. Through the above preprocessing procedures, a stable and reliable data foundation was established for subsequent high-precision keypoint detection and body measurement regression modelling.

### 2.3. Overall Framework

**Figure 3:**
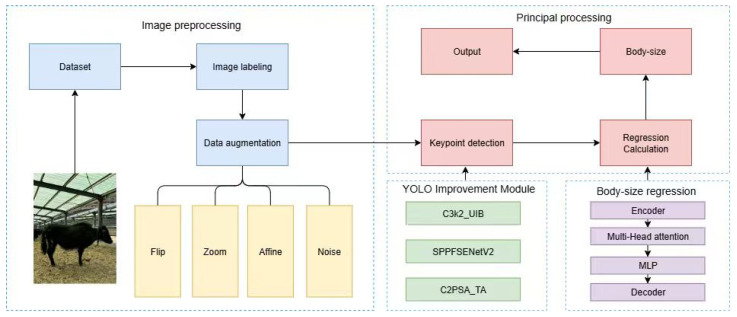
Overall framework of the proposed yak body measurement method.

The proposed intelligent yak body measurement prediction framework adopts a two-stage end-to-end modeling pipeline, as illustrated in Figure 3. First, during the data preprocessing stage, raw side-view yak images undergo keypoint annotation, scale calibration, and data augmentation to construct a high-quality training dataset. This process provides a stable input foundation for subsequent model learning.

On this basis, an improved yak body measurement prediction model, termed UST-YOLO11Pose-TRM (a YOLO11-Pose–Transformer Regression Model integrating UIB, SENetV2, and TripleAttention), is proposed. This model is consistently employed in subsequent keypoint detection and body measurement regression experiments for performance evaluation and comparative analysis.

In the main modeling stage, UST-YOLO11Pose-TRM first employs the improved YOLO11-Pose network to automatically detect keypoints on the yak body surface. This keypoint detection model enhances the original YOLO11-Pose framework by incorporating multiple attention mechanisms and is collectively referred to in this study as UST-YOLO11-Pose. The detected two-dimensional keypoint coordinates are then used to compute distance features, which are subsequently fed as input into a Transformer-based regression network.

The regression network consists of an encoder, a multi-head self-attention module, a multilayer perceptron (MLP), and a decoder. Through global feature interaction, it models the nonlinear geometric dependencies among keypoints, ultimately enabling end-to-end prediction of body measurement parameters, including body length, body height, oblique body length, chest girth, and cannon circumference.

The main advantages of this framework are reflected in the following aspects:

(1) By introducing multiple attention mechanisms at different semantic levels, hierarchical enhancement of channel, spatial, and directional features is achieved, thereby improving the robustness and stability of keypoint detection under complex poses and fur occlusion conditions.
(2) The SENetV2 module enhances the model’s adaptive modeling capability for global contextual information, thereby improving its robustness to natural plateau lighting conditions and complex backgrounds.
(3) The TripleAttention module fuses spatial and directional information, further improving the precision of keypoint localization.
(4) The Transformer-based regression model explicitly models global geometric relationships among keypoints through multi-head self-attention, thereby eliminating the need for manual feature engineering.
(5) While ensuring high prediction accuracy, the proposed model maintains a compact parameter size of approximately 10.06 MB and a computational complexity of 6.4 GFLOPs. On an NVIDIA RTX A5000 platform, it achieves an inference time of approximately 0.18 s per image, thereby meeting the real-time application requirements of smart pasture scenarios.

#### 2.3.1. Keypoint Detection Method Based on UST-YOLO11Pose

To achieve high-precision and automated extraction of yak body measurement parameters, this study proposes and adopts an improved keypoint detection model based on the YOLO11 architecture, termed UST-YOLO11Pose, for fast and stable localization of body measurement keypoints in yak side-view images. Taking preprocessed side-view yak images as input, the model automatically outputs the two-dimensional image-space coordinates of each body measurement keypoint, thereby providing reliable feature support for subsequent pixel-distance computation and Transformer-based regression modeling.

According to yak body measurement standards and image observability, a total of 10 body-surface keypoints with clear anatomical significance were selected (as shown in Figure 1). The annotated keypoints included the anterior body length point, posterior body length point, body height point, ground contact point for body height, upper chest girth point, lower chest girth point, anterior oblique body length point, posterior oblique body length point, and cannon circumference measurement points. These keypoints cover the major body measurement dimensions of yaks and are capable of comprehensively characterizing individual body conformation features.

In the process of keypoint selection, particular emphasis was placed on the biological rationality of anatomical localization, the stability of image contours, and the controllability of fur occlusion in order to enhance the robustness and generalization capability of the model under natural lighting, complex backgrounds, and diverse postures. With the synergistic enhancement of multiple attention mechanisms, UST-YOLO11Pose effectively fuses channel and spatial information, enabling precise perception of the yak body surface geometry and accurate keypoint localization.

**Figure 4:**
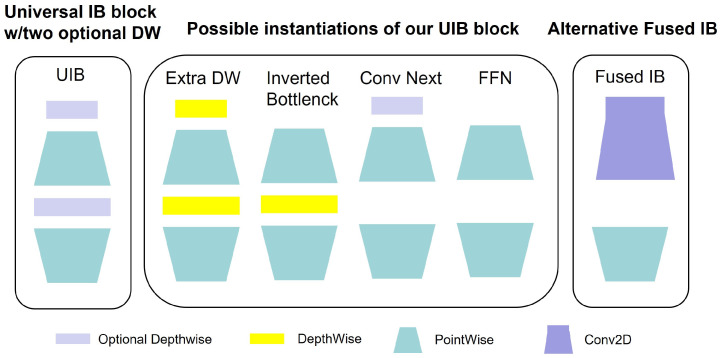
Architecture of the Universal Inverted Bottleneck (UIB) module.

**Figure 5:**
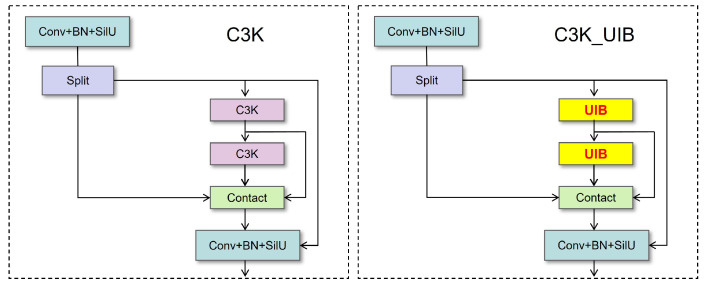
Structure of the C3k2 module integrated with the UIB mechanism.

At the network architecture level, three types of attention mechanisms are introduced into the YOLO11-Pose framework to enhance feature representation capability. First, a Universal Inverted Bottleneck (UIB) module is embedded into the C3k2 structure (as shown in Figure 4 and Figure 5). Through a design of “channel expansion–depthwise separable convolution–channel compression,” this module enhances inter-channel feature interaction while effectively controlling computational complexity. The corresponding computational process can be expressed as:

(1) $$Y=\sigma \left(W_{c}\left(\phi \left(W_{d}\left(\psi \left(W_{e}X\right)\right)\right)\right)\right),$$

where (X) denotes the input feature map; ($W_{e}$), ($W_{d}$), and ($W_{c}$) represent the expansion convolution, depthwise convolution, and compression convolution operations, respectively; and ψ and ϕ denote the nonlinear activation and normalization functions. This structure helps enhance the discriminative capability of local fine-grained features, such as the shoulder and hip keypoints.

**Figure 6:**
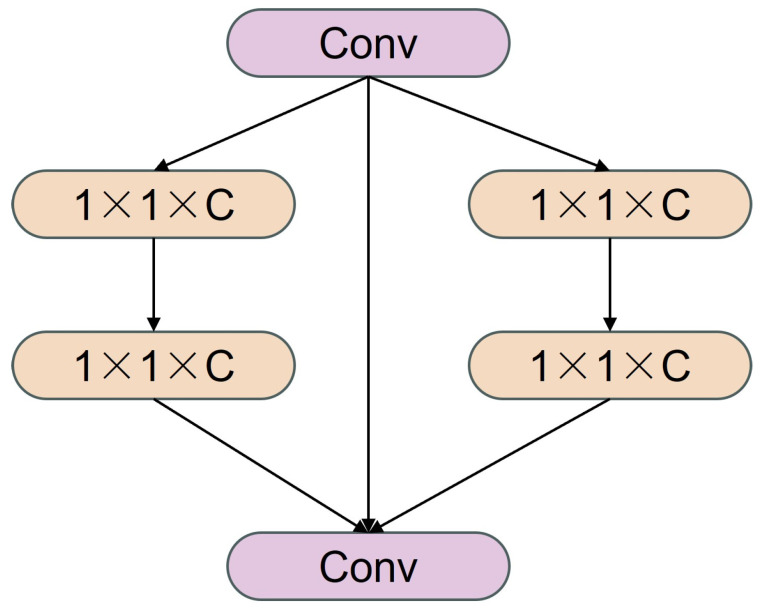
Structure of SENetV2 attention.

Second, a SENetV2 module is introduced into the SPPF structure (as shown in Figure 6). By means of global average pooling and channel recalibration, this module explicitly models global inter-channel dependencies. Its computational process is defined as follows:

(2) $$z_{c}=\frac{1}{H\times W}\sum_{i=1}^{H}\sum_{j=1}^{W}X_{c}\left(i,j\right),$$

(3) $$s=\sigma \left(W_{2}\delta \left(W_{1}z\right)\right),$$

(4) $$Y_{c}=s_{c}\cdot X_{c},$$

where $X_{c}$ denotes the c-th channel of the input feature map, H and W are the spatial dimensions, $W_{1}$ and $W_{2}$ are learnable weight matrices, $\delta \left(\cdot \right)$ represents a nonlinear activation function, and $\sigma \left(\cdot \right)$ denotes the sigmoid function. This module effectively suppresses background noise while highlighting high-response regions associated with the yak skeletal structure.

**Figure 7:**
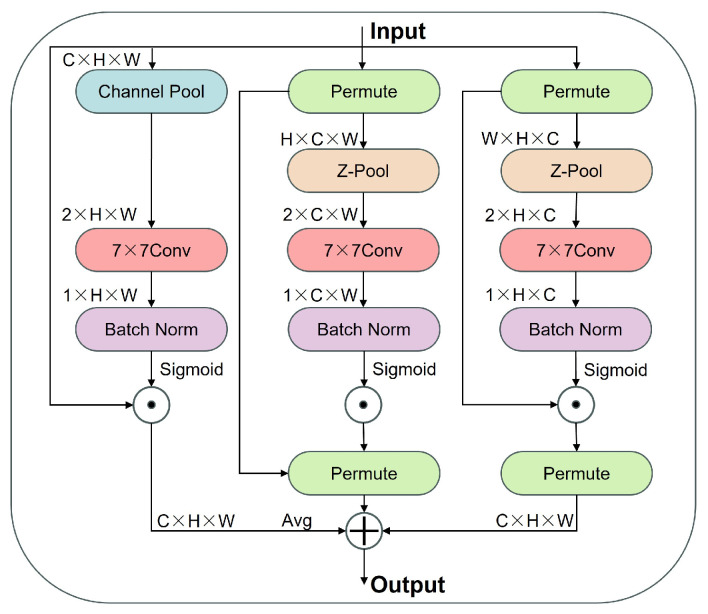
Architecture of the TripleAttention (TA) mechanism.

**Figure 8:**
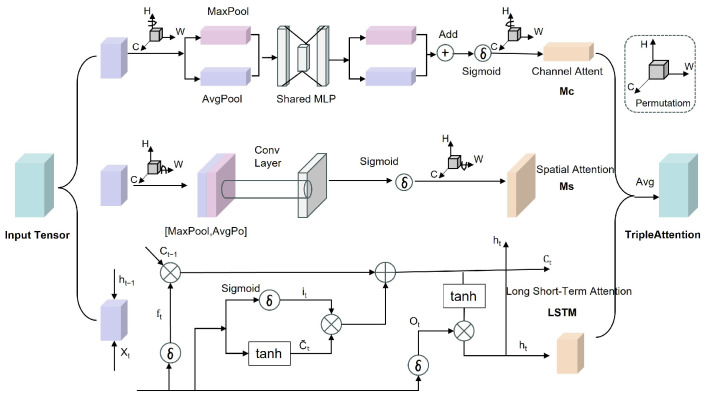
Principle illustration of the TripleAttention mechanism.

Finally, the TripleAttention (TA) mechanism is incorporated into the C2PSA structure (as shown in Figure 7 and Figure 8). This mechanism jointly models channel-wise, spatial, and directional dependencies, and its fusion form can be expressed as:

(5) $$Y=\gamma_{c}A_{c}\left(X\right)+\gamma_{s}A_{s}\left(X\right)+\gamma_{d}A_{d}\left(X\right),$$

where ($A_{c}$), ($A_{s}$), and (A_d) denote the channel, spatial, and directional attention branches, respectively. This mechanism enhances the model’s ability to perceive key structural features under varying postures.

**Figure 9:**
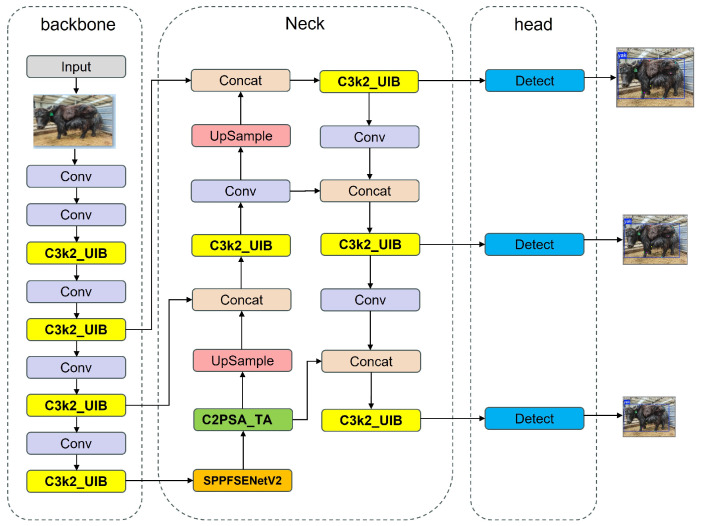
Network architecture of the proposed UST-YOLO11Pose model.

After integrating the three aforementioned attention mechanisms into the YOLO11-Pose framework (as shown in Figure 9), feature extraction is enhanced at the shallow, intermediate, and deep stages of the network. Ultimately, the model outputs the two-dimensional coordinates of yak keypoints, from which Euclidean distances are computed to construct body-surface geometric feature descriptors that serve as inputs for subsequent body measurement regression modeling.

#### 2.3.2. Transformer-Based Body Measurement Regression Method

After accurate detection of yak body measurement keypoints, a deep learning-based regression modeling module is further constructed to achieve end-to-end mapping from keypoint geometric features to actual body measurement parameters. Specifically, the two-dimensional coordinate outputs from the keypoint detection stage are first used to compute Euclidean distances, forming body-surface geometric feature vectors that describe the spatial relationships among key anatomical regions of the yak body. These feature vectors are then fed into the regression model to predict body measurement parameters, including body length, body height, oblique body length, chest girth, and cannon circumference.

To systematically evaluate the suitability of different regression architectures for the body measurement prediction task, a comparative study was conducted on four types of regression models under unified input features and training strategies. These models include a multilayer perceptron (DeepMLP), a stacked long short-term memory network (Stacked LSTM), a standard Transformer, and a Transformer with residual connections. Among them, the Transformer architecture serves as the core regression model in this study, with a particular focus on modeling global dependencies among keypoint geometric features.

The DeepMLP regression model consists of multiple fully connected layers and learns the mapping between keypoint distance features and body measurement parameters through nonlinear transformations. Although this model exhibits a relatively simple structure and stable training behavior, its capability to capture complex spatial relationships among keypoints is limited due to the absence of explicit feature interaction mechanisms. In contrast to such feedforward architectures, more advanced models are required to effectively capture global dependencies among keypoint features.

The Transformer regression model is centered on the self-attention mechanism and performs global modeling of input features through multi-head attention, enabling simultaneous focus on the spatial correlations among different keypoints. Its encoder structure can be expressed as:

(6) $$Attention\left(Q,K,V\right)=softmax\left(\frac{QK^{⊤}}{\sqrt{d_{k}}}\right)V,$$

where (Q), (K), and (V) denote the query, key, and value vectors obtained through linear projections of the input features, respectively, and (d_k) is the scaling factor corresponding to the feature dimensionality. By modeling feature relationships in different subspaces in parallel, the multi-head attention mechanism effectively enhances the model’s ability to perceive the overall structural characteristics of the yak body conformation.

Compared with conventional regression models, the Transformer offers distinct advantages for this task. Traditional methods such as MLP and CNN typically rely on local feature interactions or fixed receptive fields, which limits their ability to capture long-range dependencies among keypoints. Tree-based models (e.g., XGBoost and Random Forest) treat input features largely independently and lack explicit mechanisms for modeling structural relationships.

In contrast, the self-attention mechanism in the Transformer enables dynamic weighting of interactions among all keypoint features, allowing the model to explicitly learn global geometric relationships across the entire body structure. This is particularly important for yak body measurement prediction, where different anatomical regions are strongly correlated.

Therefore, the Transformer-based regression model provides a more suitable framework for capturing complex spatial dependencies, leading to improved prediction accuracy and better generalization performance.

On this basis, a Transformer with Residual Connections regression model is further introduced to alleviate potential gradient vanishing and feature degradation issues during deep network training. The incorporation of residual structures enhances training stability and convergence speed while preserving global modeling capability, thereby improving adaptability to variations across different body measurement scales.

The overall Transformer regression model consists of an encoder, a multi-head self-attention module, a feed-forward neural network (FFN), and a decoder. The encoder is responsible for modeling the global geometric relationships among keypoints, while the decoder projects high-dimensional features into the target body measurement parameter space through fully connected mappings, ultimately producing continuous-valued prediction outputs. This architecture eliminates reliance on manual feature engineering and enables end-to-end learning from keypoint geometric features to body measurement parameters.

During the model training stage, all regression models employed the mean squared error (MSE) as the loss function and used the Adam optimizer for parameter updates. Hyperparameter tuning and early stopping strategies were applied based on validation set performance to effectively prevent overfitting. Experimental results demonstrate that the Transformer-based regression model consistently achieves higher prediction accuracy and better fitting stability across all body measurement prediction tasks, thereby confirming its effectiveness and superiority in modeling the geometric relationships of yak body measurements.

In the regression task, the actual values refer to the ground-truth body measurement parameters obtained from manual measurements, including body length, body height, oblique body length, chest girth, and cannon circumference. The predicted values correspond to the outputs of the regression model based on keypoint-derived geometric features. Evaluation metrics such as MAE, MAPE, RMSE, and R^2^ are computed by comparing the predicted values with the corresponding ground-truth measurements.

### 2.4. Experimental Settings

All experiments in this study were conducted under the Windows 11 operating system using the PyTorch 2.0.1 deep learning framework, with CUDA 12.1 acceleration to support multi-GPU parallel training. The hardware platform comprised two NVIDIA RTX A5000 GPUs (24 GB memory each, NVIDIA Corporation, Santa Clara, CA, USA), an AMD Ryzen 9 7950X processor (Advanced Micro Devices, Santa Clara, CA, USA), and 128 GB of system memory, ensuring efficient execution of large-scale data augmentation and model training. Table 1 summarizes the main experimental configuration parameters used in this study.

All reported results are based on the final optimized models under a fixed dataset split and consistent training configuration to ensure fairness, consistency, and reproducibility. The reported metrics correspond to the final converged performance of each model. The use of a separate validation set in addition to the test set enables more reliable model selection and helps reduce the risk of overfitting compared to a simple train–test split. In addition, multiple random dataset splits were used to further evaluate the stability of the proposed method.

## 3. Experimental Results and Analysis

### 3.1. Keypoint Detection Experiments

#### 3.1.1. Intra-Series Comparison with YOLO Models

To systematically evaluate the performance advantages of the proposed UST-YOLO11-Pose in yak body measurement keypoint detection, comparative experiments were first conducted within the YOLO-series models. The comparison models included the YOLO11 series (YOLO11m, YOLO11x, and YOLO11n) as well as the latest YOLO12 series (YOLO12m, YOLO12x, and YOLO12n), all of which were comprehensively compared with the proposed UST-YOLO11Pose.

To ensure the fairness and reproducibility of the experimental results, all models were trained and tested under identical conditions, including the same dataset partitioning, data augmentation strategies, number of training epochs, optimizer settings, and hardware environment. The evaluation metrics included mean average precision (mAP), precision (P), and recall (R), as well as the number of parameters (Parameters), model size (Model Size), and computational complexity (GFLOPs).

To evaluate the performance of different keypoint detection models, experiments were first conducted at the detection stage. As shown in Table 2, the proposed UST-YOLO11-Pose significantly outperforms all comparison models across all accuracy-related metrics. It achieves an mAP of 0.958, with Precision and Recall reaching 0.967 and 0.955, respectively, demonstrating the best overall performance in the yak keypoint detection task.

Compared with YOLO11n, which exhibits the best performance within the YOLO11 series, UST-YOLO11Pose achieves a substantial improvement in accuracy while maintaining a comparable parameter scale and computational complexity. Specifically, mAP is increased by 6.4 percentage points, while Precision and Recall are improved by 7.5 and 6.9 percentage points, respectively. These results indicate that the collaborative multi-attention mechanism introduced by UIB, SENetV2, and TripleAttention effectively enhances the model’s discriminative capability for key anatomical structures on the yak body surface, demonstrating greater robustness especially in regions with severe fur occlusion and blurred edge contours.

In contrast, although YOLO11m and YOLO11x possess larger model capacities, their detection accuracy does not increase proportionally with the number of parameters. This phenomenon is mainly attributed to the relatively limited dataset size and the high sensitivity of keypoint detection tasks to fine-grained spatial localization. Under such conditions, excessively large model capacities are more prone to overfitting and fail to yield effective performance gains. Moreover, their computational overhead increases substantially (up to 202.7 GFLOPs), resulting in higher deployment costs in practical pasture environments. For the fine-grained task of yak keypoint detection, simply scaling up model size does not effectively improve performance.

Overall, the YOLO12-series models perform worse than the YOLO11 series in this experiment, particularly YOLO12m and YOLO12n, which exhibit noticeable gaps in mAP, Precision, and Recall. This indicates that the architectural design of YOLO12 is less well suited to the specific task of yak keypoint detection, whereas the YOLO11 series retains superior feature representation capability for pose and keypoint modeling.

Overall, the analysis demonstrates that UST-YOLO11Pose achieves the best balance among detection accuracy, recall capability, and model lightweight design. It not only significantly outperforms baseline models within the same series but also avoids the high computational and storage costs associated with larger models, thereby validating its strong application potential in practical yak body measurement detection scenarios.

#### 3.1.2. Cross-Model Comparison

Based on the intra-series model comparisons, to further validate the generality and advancement of the proposed UST-YOLO11Pose in the yak keypoint detection task, cross-series comparative experiments were conducted with a variety of representative keypoint detection and object detection models. The comparison models included single-stage detectors (YOLO11n, YOLO12n, and SSD), a two-stage detector (Faster R-CNN), and the high-resolution feature-preserving keypoint detection model HRNet.

All models were trained and tested under the same dataset partitioning, training strategies, and hardware environment to ensure the fairness of the experimental results. The evaluation metrics remained mAP, Precision (P), and Recall (R), while model parameter size, model footprint, and computational complexity were also jointly analyzed.

As shown in Table 3, UST-YOLO11Pose also demonstrates a significant performance advantage in cross-series model comparisons. Its mAP, Precision, and Recall all reach the highest levels—0.958, 0.967, and 0.955, respectively—clearly outperforming all comparison models.

Compared with single-stage detection models, YOLO11n achieves a certain balance between lightweight design and detection accuracy; however, its keypoint localization accuracy remains insufficient under complex yak body structures and severe fur occlusion. The overall performance of YOLO12n further declines, indicating limited suitability of its architectural design for the fine-grained task of yak keypoint detection. In contrast, UST-YOLO11Pose achieves a substantial improvement in accuracy while maintaining a computational complexity comparable to YOLO11n (6.4 GFLOPs), thereby validating the effectiveness of multi-attention mechanisms in modeling key anatomical structural features.

The two-stage detection model Faster R-CNN, which relies on a region proposal mechanism, exhibits high computational complexity (88.5 GFLOPs) and relatively poor keypoint localization accuracy, making it difficult to meet the dual requirements of real-time performance and precision in practical pasture environments. Although HRNet possesses strong high-resolution feature preservation capability, its complex network architecture and large parameter scale make it more susceptible to background interference in yak images, resulting in overall detection performance that falls short of expectations.

As a classical lightweight detection model, SSD offers certain advantages in inference speed; however, its limited multi-scale feature representation capability is insufficient for high-precision localization tasks such as yak keypoint detection, resulting in the lowest detection accuracy among the compared models.

Overall, UST-YOLO11Pose achieves an optimal balance among detection accuracy, model complexity, and computational efficiency. It not only significantly outperforms traditional two-stage and single-stage detection models but also surpasses existing YOLO-series models under lightweight constraints, thereby providing more reliable keypoint inputs for subsequent high-precision body measurement regression modeling.

### 3.2. Regression Experiments

After obtaining feature distances from the keypoint detection stage, this study further evaluated the performance of multiple regression models in predicting yak body measurement parameters. The compared models included DeepMLP, LSTM, XGBoost, Random Forest (RF), a CNN-based regression model, and the proposed Transformer-based regression model. All models were trained and validated under the same data partitioning and preprocessing strategies to ensure fairness. To ensure the fairness and comparability of the regression model comparisons, all regression experiments were conducted using the same input features derived from identical keypoint detection results. Specifically, the Euclidean distance vectors of keypoints output by UST-YOLO11Pose were uniformly adopted as the inputs for all regression models. After obtaining reliable keypoint detection results, the second stage focuses on regression modeling for body measurement estimation. Table 4 lists the performance of each model on the test set in terms of root mean square error (RMSE), mean absolute error (MAE), mean absolute percentage error (MAPE), coefficient of determination (R^2^), and model size. All evaluation metrics are calculated by comparing the predicted body measurement values with the corresponding ground-truth measurements obtained through manual measurement.

#### Regression Model Comparison

Based on the above analyses, the final integrated model combining keypoint detection and regression is evaluated to assess overall performance. As shown in Table 4, the Transformer-based regression model outperforms all other comparison methods across all evaluation metrics. It achieves an RMSE of 0.185, an MAE of 0.122, an MAPE of 2.3%, and an R^2^ of 0.962, demonstrating stronger fitting capability and better generalization performance. Compared with DeepMLP and CNN models, the Transformer exhibits a clear advantage in capturing global relationships among keypoint distance features. Compared with traditional machine learning methods such as XGBoost and Random Forest, the Transformer is able to model more complex feature interactions, thereby achieving superior performance in nonlinear mapping tasks.

It should be noted that the results reported here correspond to the final optimized Transformer model after hyperparameter tuning and training refinement. These results replace earlier preliminary experimental outcomes and ensure consistency with the metrics reported in the Abstract.

The LSTM model performs relatively poorly on this task, with both RMSE and MAPE being significantly higher than those of the Transformer and DeepMLP models. This is mainly because yak keypoint distance features do not exhibit temporal sequence characteristics, preventing the LSTM from leveraging its strength in long-term dependency modeling while also incurring higher training overhead. It should be noted that when keypoint features are fed into the LSTM network, they are arranged according to a predefined anatomical order, which does not correspond to a true temporal sequence. This arrangement further limits the LSTM’s ability to model the spatial geometric relationships of the yak body surface. Although the CNN-based model has certain advantages in local feature extraction, it remains insufficient in modeling global relationships.

Overall, the proposed Transformer-based regression model achieves the highest accuracy in body measurement parameter prediction while maintaining a moderate model size, thereby demonstrating its effectiveness in the yak body measurement prediction task.

### 3.3. Ablation Experiments

To further verify the contributions of each attention mechanism module in the proposed UST-YOLO11Pose to yak keypoint detection performance, and to analyze their synergistic relationship with the subsequent Transformer-based regression modeling, systematic ablation experiments were designed and conducted in this study. By progressively introducing different attention mechanism modules, changes in model performance were quantitatively evaluated, thereby validating the rationality of the overall network architecture design.

In the ablation experiments, five model variants were constructed, as described below: E0 denotes the baseline YOLO11-Pose model without any attention mechanisms; E1 denotes the model with the Universal Inverted Bottleneck (UIB) module introduced into the C3k2 structure; E2 denotes the model with the SENetV2 channel attention mechanism introduced into the SPPF structure; E3 denotes the model with the TripleAttention (TA) module introduced into the C2PSA structure; Ep represents the final model proposed in this study, which simultaneously integrates UIB, SENetV2, and TA attention mechanisms.

All models were trained and tested under the same dataset, training strategies, and experimental environment. The evaluation metrics included mAP, Precision (P), and Recall (R), as well as model parameter size, model footprint, and computational complexity.

To further analyze the contribution of individual components in the proposed detection model, ablation experiments were conducted. As shown in Table 5, different attention mechanisms exhibit significantly different impacts on yak keypoint detection performance. The baseline model E0, without any attention mechanisms, already demonstrates a certain level of detection capability; however, it still suffers from unstable localization in regions with severe fur occlusion and blurred body surface contours.

In the E1 model, where only the UIB module is introduced, the parameter size and computational complexity are further reduced, but the overall mAP shows a slight decrease. This indicates that the primary role of the UIB module lies in improving the efficiency and compactness of feature representations rather than independently achieving a significant boost in detection accuracy. Its performance advantages are mainly realized through synergistic interaction with other attention mechanisms. While enhancing feature representation efficiency, the UIB module contributes relatively limited improvements to detection accuracy, with its main strengths reflected in model lightweighting and feature compression.

The E2 model, which incorporates the SENetV2 channel attention mechanism, achieves modest improvements over E0 in terms of mAP, Precision, and Recall. This suggests that adaptively reweighting channel responses helps enhance the model’s ability to focus on key information on the yak body surface, providing improved robustness, particularly under varying lighting conditions and background interference.

In contrast, the E3 model, which introduces the TripleAttention module alone, exhibits a more pronounced performance improvement. Its mAP increases to 0.914, with Precision and Recall reaching 0.917 and 0.920, respectively. These results indicate that the multi-dimensional attention mechanism integrating spatial, channel, and directional information offers clear advantages in capturing the geometric structure and key anatomical features of the yak body surface, thereby effectively improving keypoint localization accuracy.

When all three attention mechanisms operate synergistically, the final model Ep achieves the best performance across all metrics. Its mAP increases to 0.958, representing a 6.4 percentage point improvement over the baseline model E0, while Precision and Recall are elevated to 0.967 and 0.955, respectively. With computational complexity remaining essentially unchanged, the detection performance is significantly enhanced, thereby validating the effectiveness of the multi-attention synergistic design.

The above analysis indicates that the three attention mechanisms introduced in UST-YOLO11-Pose exhibit strong functional complementarity. The UIB module contributes to improved feature representation efficiency and reduced model redundancy, the SENetV2 module enhances semantic discriminability at the channel level, and the TA module significantly strengthens spatial and directional feature modeling. Their synergistic integration not only improves keypoint detection accuracy but also provides more stable and discriminative keypoint inputs for subsequent Transformer-based body measurement regression modeling, thereby forming a performance-closed loop for the end-to-end intelligent body measurement prediction framework.

The ablation results further demonstrate that the performance improvements introduced by different attention modules are consistently reflected across multiple evaluation metrics, including mAP, Precision, and Recall. Moreover, a clear incremental improvement trend can be observed as more modules are incorporated into the model, indicating that each component contributes complementary benefits to the overall architecture.

Although statistical significance tests are not included, the consistency of these improvements across different metrics and configurations provides strong empirical evidence supporting the effectiveness and rationality of the proposed modules.

### 3.4. Robustness and Generalization Analysis

#### 3.4.1. Generalization Performance Evaluation

To further evaluate the generalization capability of the proposed UST-YOLO11Pose-TRM framework under diverse and challenging conditions, additional robustness experiments were conducted by introducing various perturbations to the test set.

Specifically, four types of common real-world disturbances were simulated, including (1) brightness variation (±30%), (2) Gaussian noise, (3) motion blur, and (4) random occlusion. These perturbations are designed to mimic challenging plateau pasture conditions such as strong illumination, sensor noise, motion-induced blur, and partial occlusion.

The performance of the Transformer-based regression model under these conditions is summarized in Table 6.

As shown in Table 6, although performance degradation is observed under increasing disturbance levels, the proposed model maintains relatively stable prediction accuracy across all scenarios. In particular, the R^2^ values remain above 0.93, indicating strong robustness and generalization capability.

These results demonstrate that the proposed framework is capable of adapting to a range of realistic environmental variations and retains reliable predictive performance under non-ideal conditions.

#### 3.4.2. Failure Case Analysis and Error Visualization

To further evaluate the limitations of the proposed method, a failure case analysis was conducted to investigate the impact of challenging conditions on model performance.

Several representative failure cases were identified, primarily associated with (1) complex animal postures, (2) partial occlusion, and (3) challenging lighting conditions. When yaks exhibit extreme postures (e.g., bending, turning, or partially lying down), the spatial relationships among keypoints deviate from typical configurations, leading to increased prediction errors. In cases of partial occlusion, such as overlapping body parts or obstruction by other animals, certain keypoints cannot be accurately detected, which directly affects the downstream regression results. In addition, strong illumination variations, including backlighting and shadow regions, may reduce image contrast and degrade feature extraction quality.

**Figure 10:**
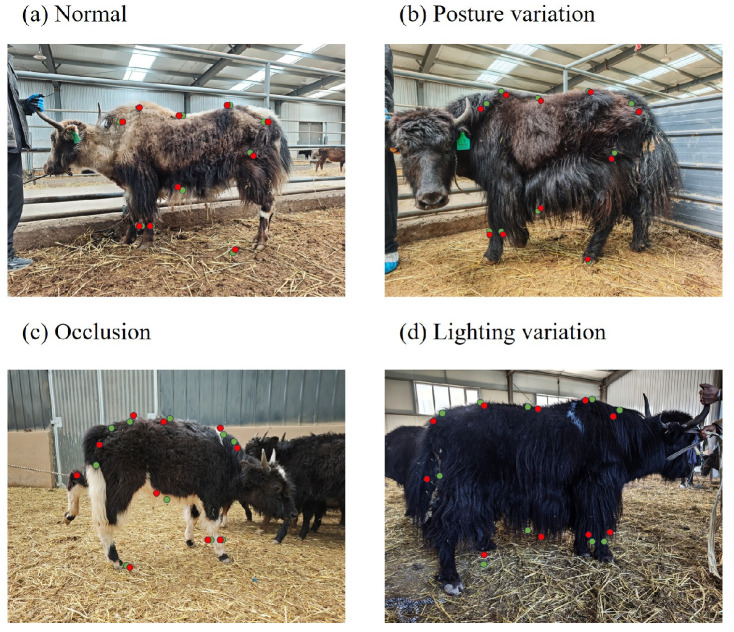
Visualization of representative prediction results under different conditions.

To visualize these effects, representative examples with relatively large prediction errors are shown in Figure 10. (Green points indicate ground-truth keypoints, and red points indicate predicted keypoints. (a) Normal condition; (b) posture variation; (c) partial occlusion; and (d) challenging lighting. Larger discrepancies between prediction and ground truth are observed under occlusion, posture distortion, and lighting variations.) The predicted keypoints and corresponding body measurements are compared with ground-truth annotations, highlighting the discrepancies under different challenging conditions.

Furthermore, a quantitative analysis was performed by grouping test samples based on their visual conditions. The average prediction errors under different factors are summarized in Table 7.

The results indicate that occlusion and complex postures have the most significant impact on prediction accuracy, while lighting variations also contribute to performance degradation. Despite these challenges, the proposed method maintains relatively stable performance across different conditions, demonstrating a certain level of robustness.

These findings provide insights into the limitations of the current model and suggest directions for future improvements, such as enhancing robustness to occlusion and incorporating more diverse training data.

#### 3.4.3. Stability Analysis Under Different Data Splits

To further evaluate the stability of the proposed method with respect to data partitioning, additional experiments were conducted using different random dataset splits. Specifically, three independent data splits were generated with different random seeds while maintaining the same training, validation, and test ratio.

The experimental results under different splits are summarized in Table 8. It can be observed that the proposed Transformer-based regression model achieves consistently strong performance across different data partitions, with only minor variations in all evaluation metrics.

These results indicate that the model is not overly sensitive to a specific data split and demonstrates stable performance under different data configurations, thereby enhancing the reliability of the proposed method.

Based on the comprehensive experimental results, a clear conclusion can be drawn: for yak body measurement keypoint detection and prediction tasks, the proposed UST-YOLO11Pose-TRM method demonstrates significant advantages in detection accuracy, model efficiency, and overall robustness.

### 3.5. Summary

Both intra-series and cross-series comparative experiments demonstrate that the multi-attention-enhanced UST-YOLO11Pose significantly outperforms YOLO11, YOLO12, and various classical detection models in key metrics such as mAP, Precision, and Recall. While maintaining a lightweight architecture and low computational complexity, it achieves high-precision localization of key anatomical structures on the yak body surface. The regression model comparison experiments further validate the effectiveness of the Transformer-based regression approach for body measurement prediction. By leveraging multi-head self-attention, it fully models the global geometric dependencies among keypoints, thereby significantly improving the accuracy of body measurement parameter prediction. Ablation experiment results indicate that the three attention mechanisms—UIB, SENetV2, and TripleAttention—exhibit strong functional complementarity, and their multi-module synergy is a key factor in enhancing keypoint detection performance and overall prediction accuracy.

Overall, UST-YOLO11Pose-TRM establishes a complete technical pipeline from high-precision keypoint detection to robust body measurement regression prediction, providing an effective solution for intelligent yak body measurement and growth monitoring that balances both accuracy and practicality.

## 4. Discussion

### 4.1. Analysis of the Improved YOLO11Pose Keypoint Detection Performance

Experimental results indicate that the proposed UST-YOLO11Pose outperforms the original YOLO11/YOLO12-series models, as well as typical two-stage and heatmap-based methods, in yak keypoint detection tasks, demonstrating superior accuracy and stability. Notably, this performance improvement is achieved without a substantial increase in network size, maintaining the model’s lightweight characteristics.

This advantage mainly stems from the introduction of multi-level attention mechanisms into the backbone network, enabling the model to more effectively focus on key structural features of the yak body surface in complex natural scenes. Unlike simply deepening or widening the network, the proposed approach enhances feature selectivity and discriminative representation, improving the model’s robustness to pose variations, occlusion, and background interference, thereby achieving stable performance even with a limited dataset.

### 4.2. Analysis of the Synergistic Effects of Attention Mechanisms

Ablation experiment results indicate that each attention module individually improves keypoint detection performance to varying degrees, while their combined use achieves the best results, demonstrating a clear synergistic effect.

Specifically, the UIB module strengthens inter-channel feature interactions, providing stable representations for low- and mid-level feature learning; the SENetV2 module introduces global context modeling, helping capture the overall body shape of the yak; and the TA module enhances spatial and directional sensitivity in high-level feature maps, improving the localization accuracy of structurally complex keypoints. The complementary roles of these three mechanisms across different semantic levels are the key factors behind the significant performance improvement of the final model.

### 4.3. Analysis of the Advantages of the Transformer-Based Regression Model

In the body measurement regression stage, the Transformer-based regression model outperforms traditional regression methods across all metrics, demonstrating higher prediction accuracy and stability. This is primarily attributable to the self-attention mechanism’s ability to explicitly model nonlinear and long-range geometric relationships among keypoints.

Unlike MLP or tree-based models that treat features as independent inputs, the Transformer can learn the latent structural relationships among keypoints related to body measurements. This allows the regression process to better reflect the geometric characteristics of the animal’s body, thereby improving the overall reliability of body measurement predictions.

Compared with alternative regression approaches evaluated in this study, the Transformer consistently achieves superior performance across all metrics. This further confirms that explicitly modeling global dependencies among keypoint features is critical for accurate body measurement prediction, and validates the suitability of the Transformer architecture for this task.

The additional generalization experiments further confirm that the proposed model maintains stable performance under various perturbations, demonstrating its robustness for practical deployment in complex plateau environments.

### 4.4. Limitations and Future Directions

Although the proposed method demonstrates strong performance, several limitations should be acknowledged.

First, the dataset used in this study is relatively small, comprising 111 individual yaks and 333 original images. Although data augmentation techniques were applied to expand the dataset to 1080 images, such approaches primarily introduce variations in appearance (e.g., scale, rotation, and noise) rather than increasing the intrinsic diversity of the underlying data distribution. Therefore, the augmented data cannot fully substitute for real-world variability, which may limit the robustness of the model when encountering unseen conditions.

Second, while the dataset was collected under natural plateau grazing conditions and includes variations in lighting, posture, and partial occlusion, it remains limited in terms of large-scale scenario diversity. In particular, high-frequency real-world conditions such as severe herd-level occlusion, extreme illumination (e.g., strong backlighting or low-light conditions), and complex multi-animal interactions are not sufficiently represented.

Third, detailed population-level information, such as age, gender, and breed variation, was not systematically recorded during data collection due to practical constraints in field conditions. This limits the ability to assess the representativeness of the dataset and may introduce potential bias in the learned model.

Fourth, all data were collected from a single geographic location under relatively consistent environmental conditions, and no cross-location or external dataset validation was conducted. As a result, the generalization capability of the model to other regions, management conditions, or imaging setups remains to be further verified.

Despite these limitations, the dataset was acquired under real grazing conditions and captures natural variations in posture and illumination, providing a practical basis for model development. The experimental results indicate that the proposed method performs reliably under similar environmental conditions.

However, the applicability of the model to more diverse populations and complex field scenarios should be interpreted with caution. Future work will focus on expanding the dataset to include more individuals from multiple regions, incorporating detailed population attributes, and conducting cross-dataset validation. In addition, integrating multi-view and multimodal data will be explored to further enhance model robustness and generalization capability under challenging real-world conditions.

## 5. Conclusions

This study addresses the non-contact intelligent measurement of yak body parameters and proposes a body measurement prediction method, UST-YOLO11Pose-TRM, which integrates improved keypoint detection with deep regression modeling. The method was systematically validated on a self-constructed yak body measurement dataset.

In the keypoint detection stage, this study introduces UIB, SENetV2, and TA attention mechanisms into the YOLO11-pose framework, resulting in a lightweight yet high-precision keypoint detection model, UST-YOLO11Pose. Experimental results demonstrate that the proposed UST-YOLO11Pose achieves superior performance in keypoint detection tasks, with an mAP of 0.958, a Precision of 0.967, and a Recall of 0.955 (Table 3). Compared with the baseline model, the mAP is improved by 6.4%, highlighting the effectiveness of the multi-attention mechanism design (Table 5).

In the body measurement regression stage, the Transformer-based model achieves an RMSE of 0.185, an MAE of 0.122, an MAPE of 2.3%, and an R^2^ of 0.962 (Table 4), demonstrating strong predictive accuracy and fitting capability. These evaluation metrics are computed based on the comparison between model predictions and manually measured ground-truth values. These results confirm that the proposed framework effectively captures the geometric relationships among keypoints and provides reliable body measurement estimation under complex natural conditions.

In summary, the proposed UST-YOLO11Pose-TRM method enables high-precision detection of yak body measurement keypoints and reliable prediction of body measurement parameters while maintaining computational efficiency, providing a feasible technical solution for growth monitoring and intelligent farming of yaks in plateau regions. Future research can further evaluate the model’s generalization ability on larger, multi-regional datasets and explore the integration of multi-view or 3D information to enhance the comprehensiveness and robustness of body measurement prediction.

However, it should be noted that this study has certain limitations. For example, the dataset is relatively small, covering only 111 yaks from a single source, and does not yet encompass the diversity of individuals from different regions or growth stages. Future research will aim to expand the data collection scope and explore multimodal fusion approaches—such as integrating nutritional information and environmental variables—to enhance the model’s generalizability and practical value.

## Figures and Tables

**Table 1:** Experimental Configuration Parameters Table.

Config	Value
Processor	AMD Ryzen 97950X
RAM	128 GB
Graphics	NVIDIA RTX A5000 × 2
Operating System	Windows 11 Deep Learning Framework
Optimizer	SGD
Batch size	16
Training epochs	200
Processor	AMD Ryzen 97950X
RAM	128 GB
Image size	640 × 640

**Table 2:** Performance comparison of different YOLO-series models for keypoint detection.

Model	mAP (%)	P	R	Parameters (MB)	Model Size (MB)	GFLOPs
YOLO11m	0.886	0.851	0.842	79.68	43.9	71.4
YOLO11x	0.876	0.844	0.836	224.18	118.3	202.7
YOLO11n	0.894	0.892	0.886	10.42	5.9	6.9
YOLO12m	0.829	0.811	0.808	10.32	5.9	6.9
YOLO12x	0.867	0.847	0.830	14.37	5.9	6.9
YOLO12n	0.853	0.834	0.829	14.51	5.9	6.9
UST-YOLO11Pose (Ours)	0.958	0.967	0.955	10.06	5.6	6.4

**Table 3:** Performance comparison of different keypoint detection models.

Model	mAP (%)	P	R	Parameters (MB)	Model Size (MB)	GFLOPs
YOLO11n	0.894	0.892	0.886	10.42	5.9	6.9
YOLO12n	0.853	0.834	0.829	14.51	5.9	6.9
Faster R-CNN	0.747	0.720	0.715	41.32	22.7	88.5
HRNet	0.752	0.728	0.723	38.64	20.5	76.2
SSD	0.735	0.710	0.705	34.12	18.9	64.7
UST-YOLO11Pose (Ours)	0.958	0.967	0.955	10.06	5.6	6.4

**Table 4:** Comparison of body measurement prediction performance among different regression models.

Model	RMSE	MAE	MAPE (%)	R^2^	Model Size (MB)
DeepMLP	0.249	0.168	6.21	0.934	5.8
LSTM	0.355	0.228	8.37	0.882	7.6
XGBoost	0.294	0.194	7.05	0.912	4.2
RF	0.318	0.207	7.68	0.901	3.9
CNN	0.272	0.179	6.53	0.927	6.5
Transformer (Ours)	0.185	0.122	2.3	0.962	6.9

**Table 5:** Ablation study results of different attention module combinations.

Model	UIB	SENetV2	TA	mAP	P	R	Parameters (MB)	Model Size (MB)	GFLOPs
E0				0.894	0.892	0.886	10.42	5.9	6.9
E1	✓			0.885	0.880	0.895	10.12	5.7	6.4
E2		✓		0.898	0.894	0.899	10.16	5.9	6.4
E3			✓	0.914	0.917	0.920	10.16	5.9	6.4
Ep	✓	✓	✓	0.958	0.967	0.955	10.06	5.6	6.4

Note: “✓” indicates that the corresponding module is included in the model configuration.

**Table 6:** Generalization performance under different perturbation conditions.

Condition	RMSE	MAE	MAPE (%)	R^2^
Original	0.185	0.122	2.3	0.962
Brightness	0.198	0.131	2.7	0.954
Gaussian Noise	0.205	0.136	3.1	0.948
Motion Blur	0.212	0.141	3.5	0.943
Occlusion	0.221	0.148	3.9	0.937

**Table 7:** Prediction error analysis under different challenging conditions.

Condition	RMSE	MAE	MAPE (%)
Normal	0.185	0.122	2.3
Posture change	0.198	0.131	2.8
Occlusion	0.214	0.142	3.4
Lighting change	0.207	0.136	3.1

**Table 8:** Performance under different dataset splits.

Split	RMSE	MAE	MAPE (%)	R^2^
Split 1	0.185	0.122	2.3	0.962
Split 2	0.191	0.127	2.6	0.958
Split 3	0.188	0.124	2.4	0.960

## Data Availability

The data that support the findings of this study are available from the corresponding author upon reasonable request.
